# Robust T1-Weighted Structural Brain Imaging and Morphometry at 7T Using MP2RAGE

**DOI:** 10.1371/journal.pone.0099676

**Published:** 2014-06-16

**Authors:** Kieran R. O'Brien, Tobias Kober, Patric Hagmann, Philippe Maeder, José Marques, Francois Lazeyras, Gunnar Krueger, Alexis Roche

**Affiliations:** 1 Centre d'Imagerie BioMédicale-Hôpitaux Universitaires de Genève, Department of Radiology, University of Geneva, Geneva, Switzerland; 2 Advanced Clinical Imaging Technogoly, Centre d'Imagerie BioMédicale-Siemens, Lausanne, Switzerland; 3 Siemens Healthcare, Siemens Schweiz AG, Renens, Switzerland; 4 Department of Radiology, Centre Hospitalier Universitaire Vaudois, Lausanne, Switzerland; 5 Centre d'Imagerie BioMédicale-Animal and Imaging Technology core, Department of Radiology, University of Lausanne, Lausanne, Switzerland; Max Planck Institute for Human Cognitive and Brain Sciences, Germany

## Abstract

**Purpose:**

To suppress the noise, by sacrificing some of the signal homogeneity for numerical stability, in uniform T1 weighted (T1w) images obtained with the magnetization prepared 2 rapid gradient echoes sequence (MP2RAGE) and to compare the clinical utility of these robust T1w images against the uniform T1w images.

**Materials and Methods:**

8 healthy subjects (29.0±4.1 years; 6 Male), who provided written consent, underwent two scan sessions within a 24 hour period on a 7T head-only scanner. The uniform and robust T1w image volumes were calculated inline on the scanner. Two experienced radiologists qualitatively rated the images for: general image quality; 7T specific artefacts; and, local structure definition. Voxel-based and volume-based morphometry packages were used to compare the segmentation quality between the uniform and robust images. Statistical differences were evaluated by using a positive sided Wilcoxon rank test.

**Results:**

The robust image suppresses background noise inside and outside the skull. The inhomogeneity introduced was ranked as mild. The robust image was significantly ranked higher than the uniform image for both observers (observer 1/2, p-value = 0.0006/0.0004). In particular, an improved delineation of the pituitary gland, cerebellar lobes was observed in the robust versus uniform T1w image. The reproducibility of the segmentation results between repeat scans improved (p-value = 0.0004) from an average volumetric difference across structures of ≈6.6% to ≈2.4% for the uniform image and robust T1w image respectively.

**Conclusions:**

The robust T1w image enables MP2RAGE to produce, clinically familiar T1w images, in addition to T1 maps, which can be readily used in uniform morphometry packages.

## Introduction

At high magnetic fields, structural whole brain T1-weighted (T1w) images are more prone to signal variations due to presence of larger inhomogeneity of the static magnetic field (ΔB0), the transmit (B1^+^) and the receive (B1^−^) radio frequency field inhomogeneities [1]. These signal variations create an intensity bias, which could lead to misleading clinical diagnosis, incorrect gray and white matter segmentation or poor co-registration to other acquired datasets.

**Figure 1:**
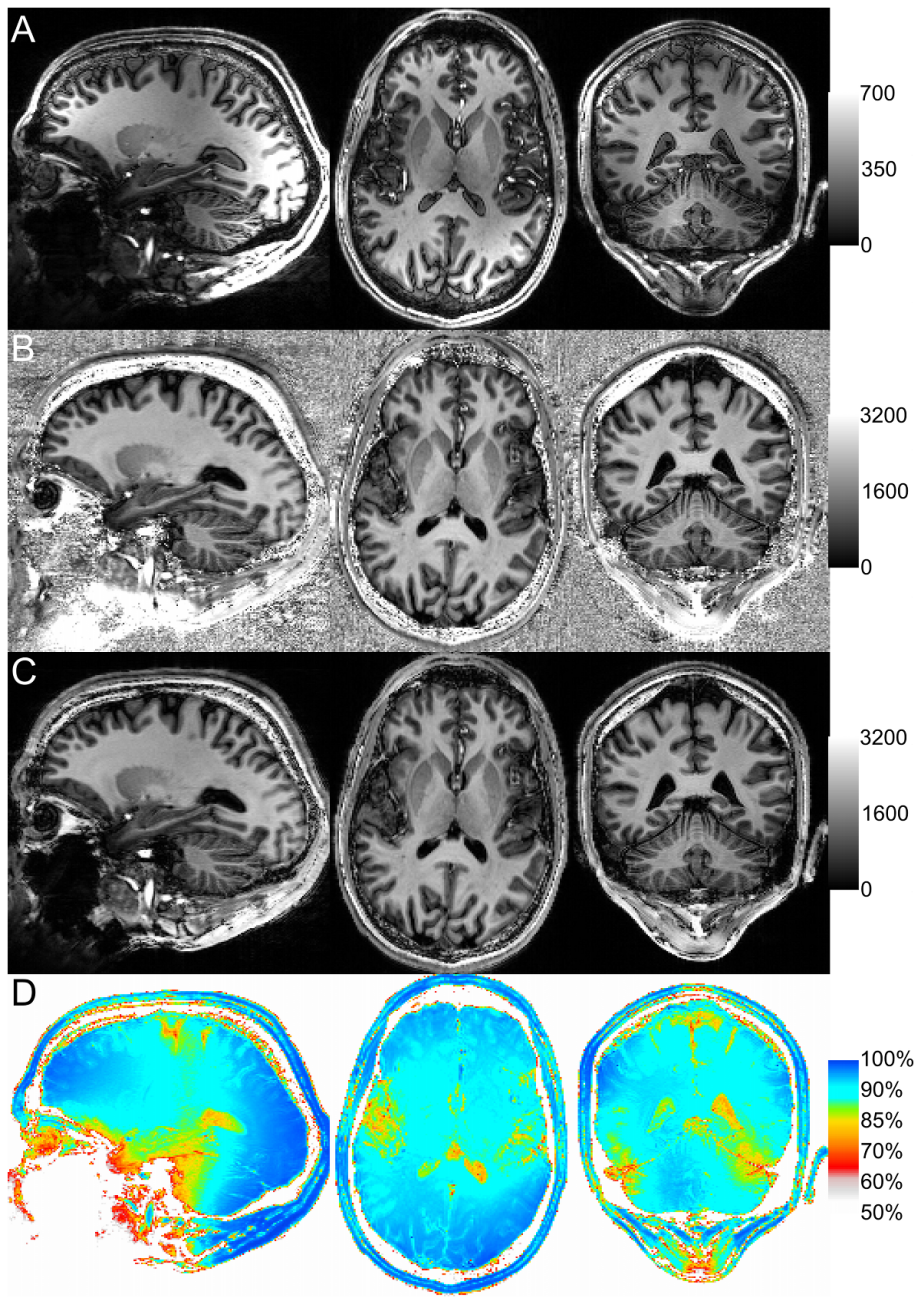
MPRAGE (A), uniform T1w (B) and robust T1w (C) MP2RAGE image volumes of a healthy volunteer (male, 31years); the windowing for each image was chosen according to the transverse images. The ratio (D = C/B) of the robust T1w over uniform T1w image shows that the inhomogeneity introduced by including β. The gross changes in the uniform T1w value occur in regions of poor B1^+^ transmit (cerebellum) or B1^−^ receive (brain centre) field coverage which result in a low signal intensity relative to β. The contrast seen in these ratio images also indicates that the choice of imaging parameters also has an effect, with cerebrospinal fluid experiencing a greater change relative to adjacent white or gray matter.

3D T1w images are often acquired with a magnetization prepared rapid acquisition gradient echo (MPRAGE) [2], [3]. The T1 contrast becomes dominated by the adiabatic radio frequency pulse used to invert the spins. However, intensity field variations from the inhomogeneous B1^−^ field remain and to a lesser extent B1+ inhomogeneities due to the small flip angles used for excitation [4]. At lower fields, these variations are often removed in post processing [5] but at higher fields (>3T) the B1+ and B1^−^ field variations are more pronounced [6]. These large signal variations (see Figure 1A) are no longer well corrected by the current intensity bias correction algorithms [4], [7].

To overcome this problem at higher magnetic fields, Van de Moortele *et al* [4] and Marques *et al* [7] proposed to take the ratio of 3D T1w MPRAGE (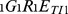) and 3D GE-PD (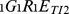) proton density weighted image volumes. In addition to the estimation of a quantitative T1map, the ratio of the two image volumes was shown to correct for the proton density, T2* contrast, and B1 inhomogeneities [4], [7]. This so-called “uniform image” exhibits better T1w contrast at the expense of amplifying noise (Figure 1B). Apart from being visually displeasing, the increased noise level in the background and cavities of the skull makes delineation of structures difficult and is problematic for registration and automatic segmentation algorithms. In post-processing the background noise could be removed by either applying a binary mask [4] or an image filter (median, Gaussian or total variation). Though effective, these approaches risk thresholding out or smoothing over image features from inside the brain.

In this work, we propose a simple modification to the normalized complex ratio that suppresses the noise in the uniform T1w image. The radiological value of the robust T1w image was qualitatively compared with the uniform T1w image by two experienced radiologists. The utility of the robust images for morphometry is demonstrated with SPM8 [8], a voxel-based morphometry package, and a volume-based morphometry package MorphoBox [9], [10].

## Materials and Methods

### Image acquisition

8 healthy subjects (29.0±4.1 years; 6 Male) underwent two identical scan sessions within a 24 hour period, the study was approved by the local ethics committee (Commission cantonale VD d'éthique de la recherché sur l'être humain) and each subject provided written consent. The uniform T1w image volumes were obtained through the use of the commercially available Magnetization Prepared with 2 Rapid Gradient Echoes (MP2RAGE) sequence [7] modified to improve the inversion coverage for whole brain acquisitions at 7T [11]. Typical image parameters were TR_MP2RAGE_/TR_READOUT_/TE 6 sec/6.5 msec/2.89 msec α_1_/α_2_ 4°/5° TI_1_/TI_2_ 0.8 sec/2.7 sec Matrix 256×240×176, voxel 1.0×1.0×1.2 mm. To show the larger B1 inhomogeneities present at 7T, 1 subject was additionally acquired with an MPRAGE sequence using identical image parameters except for the inversion time, which was set to TI 1.5 s [4]. All subjects were scanned on a Siemens Magnetom 7T head-only scanner (Siemens Healthcare Sector, Germany) with a single-channel transmit and 32 channel receive volume coil (Nova Medical Inc, MA, USA).

### Reconstruction of the uniform T1w images

The MP2RAGE sequence simultaneously acquires the T1w (GRE_TI1_) and PDw (GRE_TI2_) image volumes. The uniform T1w image volume is obtained by taking the real component of the normalized complex ratio from the two acquired image volumes (8): 
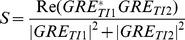
(1)


This form of the image ratio has the advantageous property of limiting the image intensity within a predefined range (−0.5 to 0.5). The amplification of the noise in the uniform T1w image is a result of the numerical instability when the denominator tends towards zero. This, added to the fact that the phase points in any arbitrary direction when the SNR is very low [12] means that the noise takes on a “salt and pepper” characteristic, spreading across the range of −0.5 to 0.5.

The numerical instability can be removed by adding a constant real number β to equation 1:
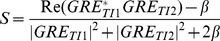
(2)


**Figure 2:**
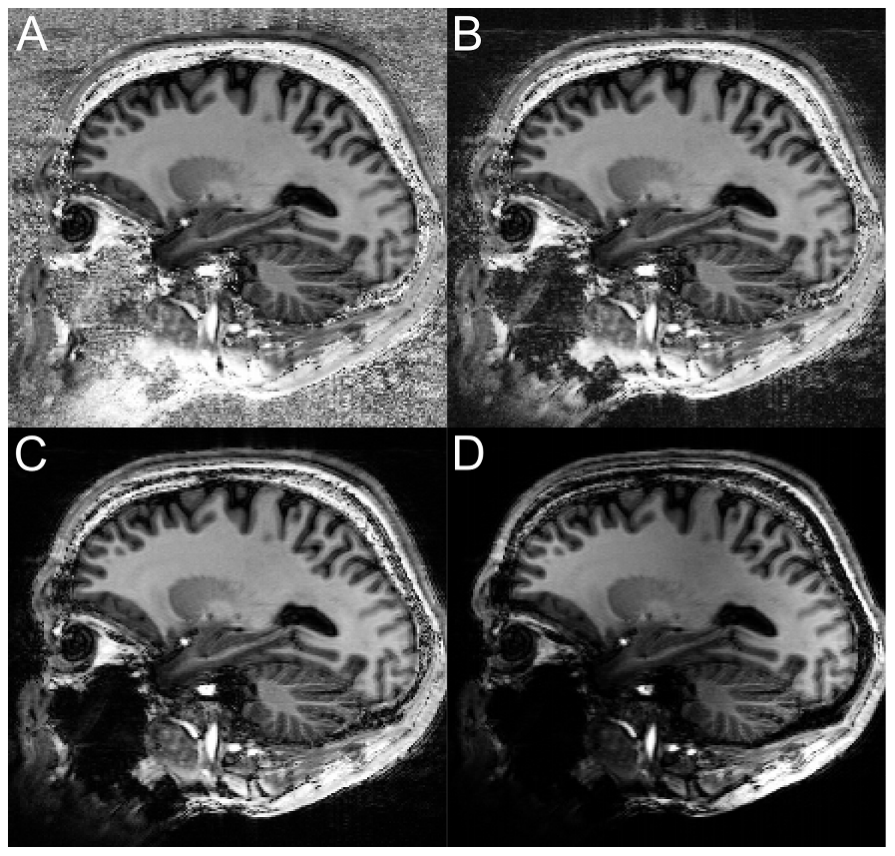
A uniform T1w MP2RAGE image (A) of a healthy volunteer (male, 31years) versus three different robust T1w images calculated with increasing β: β/10 (B) β (C) and 10β (D). β must be chosen so that adequate noise suppression occurs without introducing the significant signal intensity bias seen in D.

When the signal is very low or noise, the constant β should dominate the calculation forcing the ratio (S) to an intensity value of −0.5, which ensures that the background is darker than the cerebrospinal fluid. When the signal is large β should have minimal impact on the ratio calculation to avoid reintroducing a significant bias back into the uniform T1w image, Figure 2. The choice of β can be optimized automatically [13], or to vary dependent on the mean signal strength or the noise level of the image volume; however in practice, limited gain is achievable compared to the fixed empirically optimized β applied herein.

The uniform (equation 1) and robust (equation 2) T1w image volumes were calculated inline on the scanner.

### Image assessment

Figure 1 visually shows the improvement of the robust T1w image; however, we know that this comes at the expense of an introduced intensity bias across the images. Therefore two experienced radiologists (PH/PM) familiar with the MP2RAGE contrast were asked to qualitatively assess, from a clinical perspective, the image quality regionally over the brain. The anonymized and randomized images were rated for: i) general image quality, in line with the quality rating procedure employed in ADNI [14], ii) 7T specific artifacts including residual inversion, susceptibility and intensity inhomogeneity, and; iii) the definition of local structures including the hippocampus, thalamus, striatum, pituitary gland, and the temporal and cerebellar lobes. The scoring system was based on the ADNI rating system: 0 = severe, 1 = moderate, 2 = mild and 3 = none [15]. A positive sided Wilcoxon signed rank test was performed on the average score difference (robust – uniform) across categories for each observer assuming that the observations over subjects and repetitions can be considered independent.

**Figure 3:**
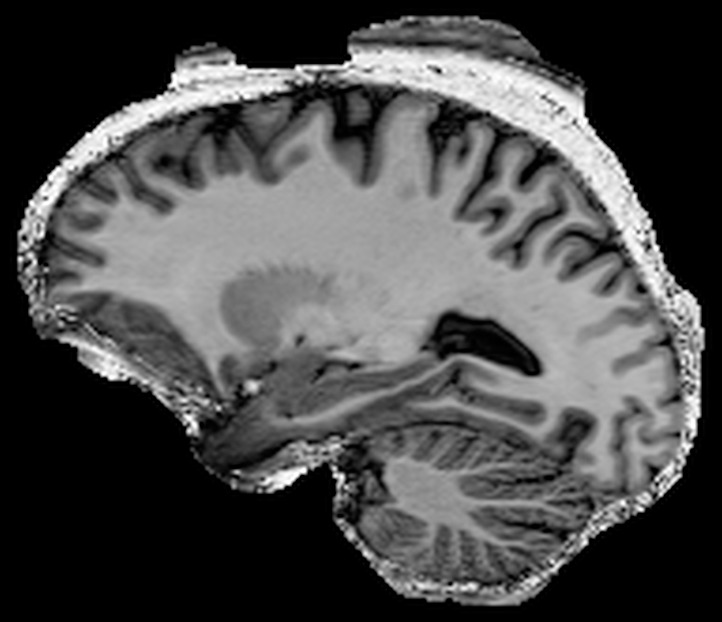
An example of the background removal that results from the pre-processing steps proposed in [16], large portions of non-brain tissue remain.

To demonstrate the utility of the robust T1w image for morphometry packages, the image volumes were segmented with SPM8 and MorphoBox. The segmentation quality between the uniform and robust images was visually assessed. The reproducibility of MorphoBox's volumetric estimates of the brain's structures between repeat scans was used to compare the segmentation quality of the uniform verse robust T1w image. The average relative volumetric difference and the worst-case errors are reported. The worst-case error was defined for each subject as the maximum across structures of relative volumetric differences between repeats in absolute value. A positive Wilcoxon signed rank test was performed on the difference of worst-case reproducibility error between the uniform and robust segmentations. The same was done between the pre-processed and robust segmentations. In addition the proposed pre-processing step for MP2RAGE images proposed in [16] was also applied, Figure 3, using the software package [17]. Here the background noise is removed by creating a mask based on the GRE_TI2_ magnitude image, followed by a region growing algorithm and level set smoothing to finally yield a skull-stripped image volume.

## Results

Figure 1 shows that the intensity inhomogeneities observed in uniform T1w MPRAGE images (Figure 1A) can be overcome using the recently introduced MP2RAGE sequence (Figure 1B). This correction comes with a significant amplification of the noise in the image background that can be efficiently suppressed by substituting equation 1 for equation 2 (Figure 1C) with only minimal differences in signal intensity and contrast (Figure 1D).

**Figure 4:**
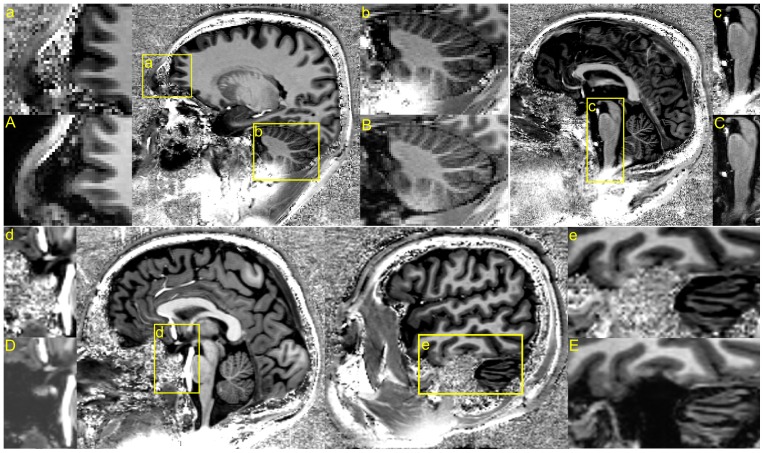
Zoomed examples comparing uniform and robust T1w MP2RAGE images from healthy volunteers showing the noise suppression inside the skull (A); improved delineation of the cerebella lobes (B), brain stem (C), pituitary gland (D) and the temporal lobe (E) through the removal of the background noise and compensation of the inversion artefact.

The images were judged to be of good general image quality which was reproducible between scans and observers. Table 1 shows, as expected, that the uniform image had a better qualitative rating by the radiologists for intensity inhomogeneity than the robust image. The visible inhomogeneity introduced, however, was ranked as mild. For the central structures, Thalamus and Striatum, no difference was observed but the delineation of the cerebella lobes and pituitary gland were considered superior in the robust T1w image, Figure 4. The positive sided Wilcoxon rank test showed that the robust T1w image significantly scored higher than the uniform image for both observers (p-value  = 0.0006 and 0.0004 for observer 1 and 2 respectively).

**Table 1:** Image quality scores for the uniform and robust T1w MP2RAGE image averaged across subjects for each observer and repetition.

		Observer 1				Observer 2			
		Uniform		Robust		Uniform		Robust	
		rep 1	rep 2	rep 1	rep 2	rep 1	rep 2	rep 1	rep 2
**General**	Blurring/Ghosting/Ringing	1.75	1.75	1.75	1.75	2.13	1.88	2.13	1.88
	Flow/Linear	2.88	2.75	2.88	2.75	2.50	2.00	2.50	2.00
	Wrap	2.88	2.88	2.88	3.00	2.75	2.38	2.75	2.38
	Head coverage	3.00	2.75	3.00	2.75	3.00	2.88	3.00	2.88
**7T**	Inversion	1.63	2.00	2.38	2.63	1.75	2.13	2.00	2.50
	Susceptibility	0.75	0.25	1.50	1.13	1.13	1.50	1.38	1.63
	Inhomogeneity	3.00	3.00	2.13	2.00	3.00	2.63	2.63	2.63
**Local structure definition**	Hippocampus (L)	1.75	2.13	1.88	2.13	2.63	2.75	2.63	2.88
	Hippocampus (R)	1.75	2.50	1.88	2.50	2.88	2.88	2.88	3.00
	Thalamus (L)	3.00	3.00	3.00	3.00	3.00	3.00	3.00	3.00
	Thalamus (R)	3.00	3.00	3.00	3.00	3.00	3.00	3.00	3.00
	Striatum (L)	3.00	3.00	3.00	3.00	3.00	3.00	3.00	3.00
	Striatum (R)	3.00	3.00	3.00	3.00	3.00	3.00	3.00	3.00
	Pituitary	0.38	0.75	1.50	1.88	0.00	0.00	0.75	1.00
	Cerebella lobes (L)	1.38	1.38	2.25	2.00	1.88	2.00	2.00	2.38
	Cerebella lobes (R)	1.38	1.38	2.25	2.00	2.25	2.50	2.25	2.75
	Temporal (L)	1.38	1.88	1.75	2.00	0.75	2.00	0.88	2.13
	Temporal (R)	1.38	1.88	1.75	2.00	0.14	1.00	0.88	1.13
	Brain Stem	2.38	2.25	2.38	2.25	1.88	2.00	2.13	2.13

**rep**  =  repetition, Scores: 0 = severe, 1 = moderate, 2 = mild and 3 = none.

**Figure 5:**
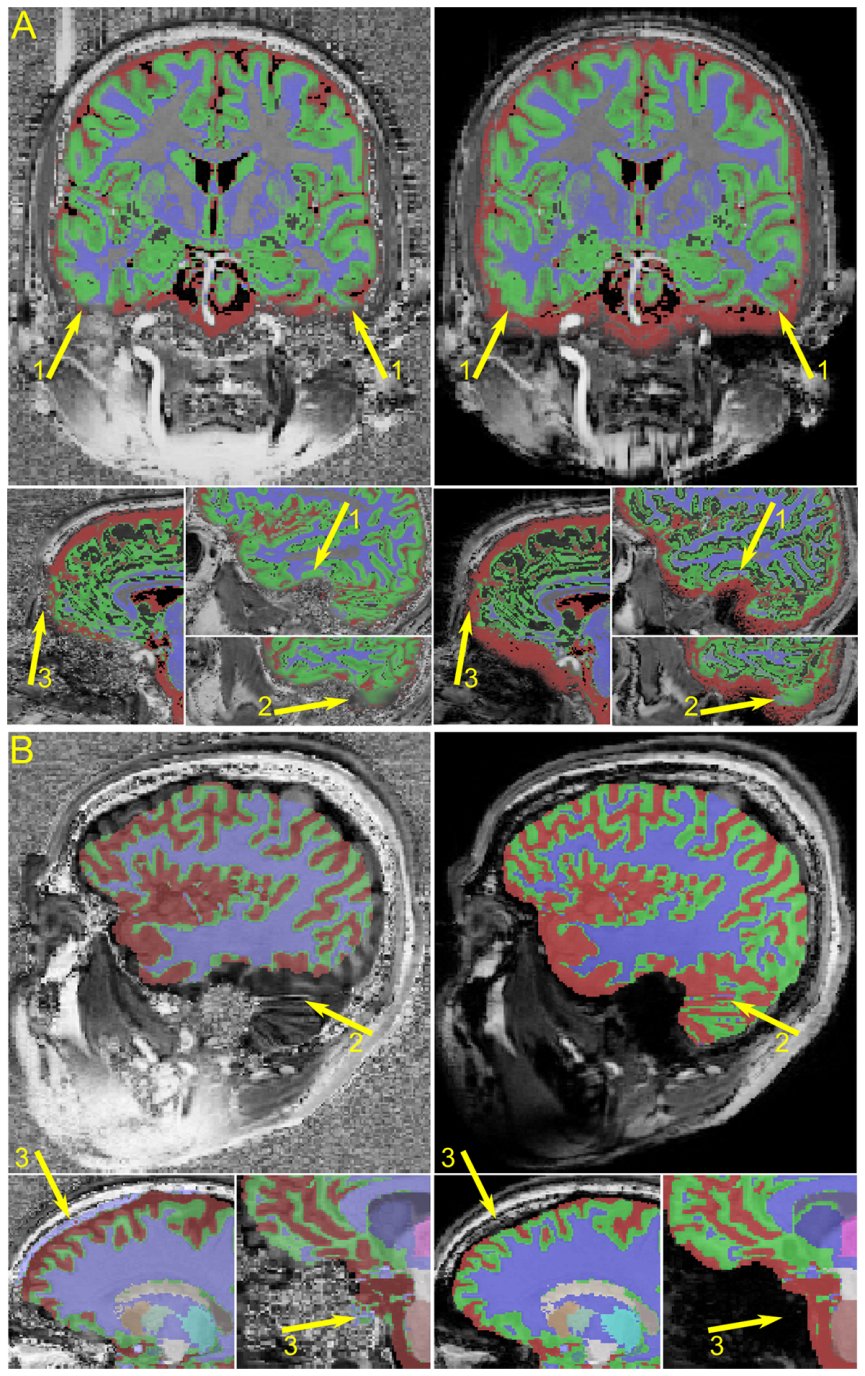
Examples of the segmentation results with SPM8 (A) and MorphoBox (B) using uniform and robust T1w MP2RAGE images of healthy volunteers. The colours represent the probability map from SPM8 or the label map from MorphoBox: red  =  cerebrospinal fluid; blue  =  white matter; green  =  gray matter; and the other colours represent additional structures identified with MorphoBox. The arrows indicate areas of more consistent segmentation with the anatomy (1); missed structures (2); and, background noise misclassified as either gray or white matter (3).

Figure 5A shows examples of segmentation results using SPM 8 and MorphoBox. With the SPM8 processing, the cerebrospinal fluid in the uniform T1w image often appeared underestimated compared to the robust T1w image. Differences were also observed in the temporal and frontal lobes where the background noise of the uniform T1w image either interfered with the distinction of the cortex's boundary or had a high probability to be considered gray matter. In addition, it was often observed that the parts of the cerebellar lobes were missed by the segmentation. With the MorphoBox the fatty tissue inside the parietal bone could also be misclassified as white matter on the uniform image without background noise suppression.

**Figure 6:**
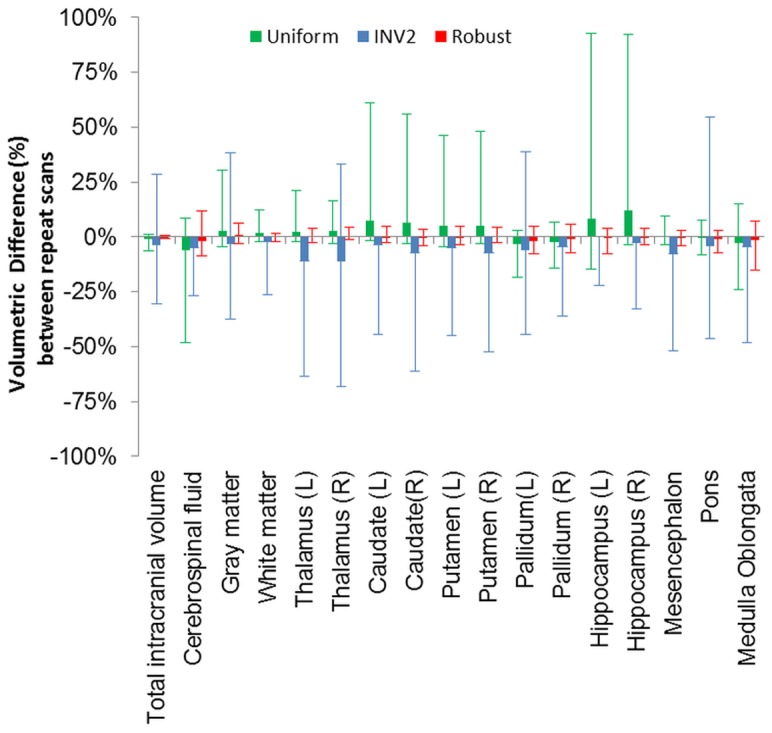
Percentage differences between repeat scans of the segmentation results when utilizing the uniform and robust T1w image. (Error bars  =  max/min).

The reproducibility of the volumetric estimate of the various structures segmented by MorphoBox significantly improved (p-value = 0.043/0.039) between repeat scans when the robust T1w images are used as input compared to the uniform T1w image without and with preprocessing step. With the uniform image, even with the proposed pre-processing step, large variations can occur, Figure 6. For example the volume of the hippocampus in one subject differed by 93% between scans due to the complete failure in one of the volumes of the registration of the initial atlas and subsequent tissue misclassifications. With the robust image the variation in the same subject was only 2.8%. On average the difference between volumetric estimates across all 25 processed structures is ≈6.6% for the uniform image without preprocessing and ≈18.2% with the preprocessing step, compared to ≈2.4% for the robust T1w image. The worst-case reproducibility error averaged across subjects yielded ≈21.4% for the uniform image without preprocessing and ≈32.7% with the preprocessing step, compared to ≈8.5% for the robust T1w image.

## Discussion

The proposed modification to the MP2RAGE calculation is a tradeoff between the self-bias correcting properties of the ratio and numerical stability. The empirically determined β effectively suppresses the noise inside and outside the skull without affecting image features within the brain. The small bias introduced does not affect the reproducibility of segmentation results and the radiological assessment of the MP2RAGE images found that the removal of the background noise and the improved delineation of specific structures e.g. cerebellar lobes, temporal lobes and in particular the pituitary gland also outweighed this introduced signal inhomogeneity. Visually the robust images appear more like customary T1w MPRAGE images.

With MorphoBox it was clear that the robust T1w image enabled a more robust and reproducible segmentation. The most likely cause of segmentation failure with MorphoBox was the initial registration of an atlas used to skull strip the data. Large portions of the brain were often cut out with the uniform compared to the robust T1w image. Similarly the pre-processing steps outlined in [16] inadequately skull stripped the data leaving large portions of the background noise in the image, Figure 3. On the other hand SPM8, which utilizes a probabilistic atlas to simultaneously solve the registration and tissue classification steps, avoids large registration errors but remained prone to misclassification artifacts when structures bordered regions of noise in the uniform versus the robust T1w image, Figure 5.

In addition to noise suppression and improved segmentation, the robust image apparently compensated the bright signal inversion artifacts experienced at 7T due to poor B1^+^ coverage [11] over the cerebellum and brain stem. The preference of each observer for the robust image quality was due to an apparent recovery of some contrast in these regions. Unfortunately the inversion artefact remains in both images. Only the way it manifests itself in the image is different.

In the uniform image, equation 2, in regions of poor inversion the signal intensities of both images become approximately equal. Equation 1 thus heads towards 0.5 and we obtain the regions of bright signal intensity. In the robust image, equation 2, these regions of poor B1^+^ coverage appear to recover contrast because their signal intensities are of similar magnitude to β allowing the subtle difference from the different flip angles to appear more pronounced. The signal intensities are spread out or stretched, similar to histogram equalization [18].

The removal of artificial bright signal intensity regions in the robust T1w image is advantageous for registration and helps clearly distinguishes the blood vessels who also appear bright due to the fresh inflow of unsaturated spins at 7T [4]. For image registration, the regions of bright signal intensity may cause large local intensity discrepancies between the target and source image. The discrepancy could interfere with the registration algorithm's measure of similarity and result in poorly aligned images. However, the quantitative T1 maps proposed in [7] should still be based on the uniform T1w image and the segmentation results need to account for the change in T1 contrast in order to maintain accuracy in these regions.

Similarly, observer 1 rated the susceptibility artifacts differently between the robust and uniform T1w images; however, the susceptibility induced signal loss remains unchanged, Figure 4e. In the uniform T1w image the regions of susceptibility induced signal loss manifest as salt and pepper noise. In this example of Figure 4e, bright signal intensities are observed inside the cortex. Whilst in the robust T1w image it appears as a signal loss, similar to MPRAGE.

In this work, practical implementation issues meant β was empirically chosen based on the optimization results from [13]. The use of a fixed β was sufficient in this study because the image parameters and, as detailed in [11], the reference voltage was held fixed across subjects: however, in a clinical scenario this may not be sufficient. Future work is required to either incorporate the automatic optimization inline or to test the effectiveness of the noise suppression of different values of β when image parameters and/or the reference voltage are varied. Furthermore comparison data from the morphometry of 3T MPRAGE data is required to further verify the MP2RAGE based morphometry results. A larger study is now being planned.

In summary, MP2RAGE, in addition to the provision of fast high resolution T1 maps, can produce clinically familiar T1w images at 7T through a simple modification of the image ratio calculation to suppress the amplification of the noise. The robust T1w MP2RAGE image is shown to outperform the uniform T1w MP2RAGE image in terms of radiological value (as evaluated by experienced clinicians) and enables reproducible volumetry results (as evaluated by automatic segmentation packages) without any additional pre-processing.
